# Mutational analysis of SARS-CoV-2 ORF6-KPNA2 binding interface and identification of potent small molecule inhibitors to recuse the host immune system

**DOI:** 10.3389/fimmu.2023.1266776

**Published:** 2024-01-12

**Authors:** Muhammad Suleman, Afsheen Said, Haji Khan, Shoaib Ur Rehman, Abdulrahman Alshammari, Sergio Crovella, Hadi M. Yassine

**Affiliations:** ^1^ Laboratory of Animal Research Center (LARC), Qatar University, Doha, Qatar; ^2^ Center for Biotechnology and Microbiology, University of Swat, Swat, Pakistan; ^3^ Department of Biotechnology, University of Science and Technology, Bannu, Pakistan; ^4^ Wilhelm Johansen Centre for Functional Genome Research, Department of Cellular and Molecular Medicine, The PANUM Institute, University of Copenhagen, Copenhagen, Denmark; ^5^ Department of Pharmacology and Toxicology, College of Pharmacy, King Saud University, Riyadh, Saudi Arabia; ^6^ Biomedical Research Center, Qatar University, Doha, Qatar; ^7^ College of Health Sciences-Qatar University (QU) Health, Qatar University, Doha, Qatar

**Keywords:** SARS-CoV-2, ORF6, KPNA2, molecular docking, MD simulation

## Abstract

Severe acute respiratory syndrome coronavirus 2 (SARS-CoV-2) surfaced on 31 December, 2019, and was identified as the causative agent of the global COVID-19 pandemic, leading to a pneumonia-like disease. One of its accessory proteins, ORF6, has been found to play a critical role in immune evasion by interacting with KPNA2 to antagonize IFN signaling and production pathways, resulting in the inhibition of IRF3 and STAT1 nuclear translocation. Since various mutations have been observed in ORF6, therefore, a comparative binding, biophysical, and structural analysis was used to reveal how these mutations affect the virus’s ability to evade the human immune system. Among the identified mutations, the V9F, V24A, W27L, and I33T, were found to have a highly destabilizing effect on the protein structure of ORF6. Additionally, the molecular docking analysis of wildtype and mutant ORF6 and KPNA2 revealed the docking score of - 53.72 kcal/mol for wildtype while, -267.90 kcal/mol, -258.41kcal/mol, -254.51 kcal/mol and -268.79 kcal/mol for V9F, V24A, W27L, and I33T respectively. As compared to the wildtype the V9F showed a stronger binding affinity with KPNA2 which is further verified by the binding free energy (-42.28 kcal/mol) calculation. Furthermore, to halt the binding interface of the ORF6-KPNA2 complex, we used a computational molecular search of potential natural products. A multi-step virtual screening of the African natural database identified the top 5 compounds with best docking scores of -6.40 kcal/mol, -6.10 kcal/mol, -6.09 kcal/mol, -6.06 kcal/mol, and -6.03 kcal/mol for tophit1-5 respectively. Subsequent all-atoms simulations of these top hits revealed consistent dynamics, indicating their stability and their potential to interact effectively with the interface residues. In conclusion, our study represents the first attempt to establish a foundation for understanding the heightened infectivity of new SARS-CoV-2 variants and provides a strong impetus for the development of novel drugs against them.

## Introduction

SARS-CoV-2 (severe acute respiratory syndrome corona virus 2), was reported as an etiological agent causing a worldwide pandemic of covid-19 pneumonia-like disease, which emerged in Wuhan, China on 31 December, 2019 (1). According to the latest updates, as of 15 April 2022, the global confirmed cases are about 504 million and 6,197,159 deaths. The disease symptoms range from mild to acute, though cases with no symptoms have also been documented (2). SARS-CoV-2 is a positive-sense, enveloped, single-stranded RNA virus belonging to the Riboviria kingdom, Nidovirales order, coronaviridae family, Betacoronavirus Genes and SARS-related coronavirus species (3). The viral genome of approximately 30 kb in size consists of a 5’ and 3’ untranslated region (4). The 5’ end of the genome contains the genetic information for 16 nonstructural proteins (Nsp1-Nsp16) (5), while the 3’ end encodes 4 structural proteins (M, N, S, E) and 8 accessory proteins (ORF3a, ORF3b, ORF6, ORF7a, ORF7b, ORF8, ORF9b, ORF10) (6, 7). The NSPs (nonstructural proteins) help in replication, structural proteins are accountable for virion formation while accessory proteins are responsible for virus-host interaction, facilitating pathogenesis, infection, and *in vitro* viral replication (8).

The type 1 interferon pathway serves as the initial defense mechanism of the host’s innate immune response against viral infections. In the case of coronavirus, the virus produces double-stranded RNA (dsRNA) that is detected by pattern recognition receptors (PRRs). This recognition event leads to the activation of IRF3 (IFN regulatory factor-3), triggering a cascade of immune responses (9). Phosphorylated IRF3 undergoes dimer formation followed by nuclear translocation, activation of IFN-I genes, and stimulation of the secretion of interferon α/β (10, 11). Interferon plays a vital role against viral infection by inducing antiviral activities (12). IFNARs (interferon receptors) are activated by secreted interferon α/β that induce activation of STATI and STAT2 (13). STAT1 and STAT2 interact with IRF9 to form the ISGF3 complex, which translocates into the nucleus, and stimulates the activation of many interferons stimulated genes (ISGs) by binding with ISREs that ultimately elicit an efficient antiviral response (14).

Corona-virus developed diverse strategies to counteract the IFN pathway and to antagonize the IFN response by targeting distinct steps in the IFN production pathway (15). Among SARS CoV-2 accessory proteins, ORF6 (accessory protein open reading frame 6) is a small polypeptide of about 7-kDa that is composed of 61 amino acids, shows 69% sequence similarity with ORF6 of SARS-CoV, and has been exhibited to antagonize host antiviral responses and also contributes in viral infection pathogenesis. ORF6 protein targets the interferon production pathway by binding with karyopherin (KPNA2). KPNA2, encodes importin alpha 1, to which ORF6 could bind. Several levels of regulation take place at nuclear import of the ISGF3 complex. Normally, the ISGF3 complex (activated STAT1) exposes NLS (nuclear localization signal) on its surface, recognized by KPNA1 which recruits KPNB1 for nuclear transport of complex (ISGF3:KPNA1) via nuclear pore (16). In SARS-CoV-2 infected cells ORF6 is present at the Golgi apparatus/Endoplasmic reticulum membrane. The ORF6 C-terminal amino acids directly interacted with KPNA2 which recruits KPNB1 from the cytoplasm to the membrane complex and causes the depletion of free unbound KPNB1 consequently, restraining nuclear transport of ISGF3 complex. ORF6 binding to KPNA2 indirectly block the transport of ISGF3:KPNA1 into the nucleus leading to the inhibition of STAT1 nuclear translocation resulting in the suppression of the interferon pathway (17). ORF6 also restrains IFNβ production through binding with import factor KPNA2, inhibiting IRF3 nuclear transport (18). Taken together, ORF6 binding with KPNA2 inhibits the nuclear transport of STAT1 and IRF3, resulting in the suppression of the host immune system.

Various studies reported that ORF6 antagonizes the IFN production pathway to escape human immune response through interaction with the KPNA2 complex (17, 18). Since various mutants were therefore emerging, it is important to explore whether these mutants counteract IFN production and promote the pathogenesis of viral infection by altering the structure stability and binding affinity of ORF6 with KPNA2. In the present study, we used biophysical analysis and comparative binding techniques to reveal the effect of newly emerged and deleterious mutations in ORF6 on immune evasion by physically interacting with KPNA2. The binding interfaces of ORF6 and KPNA2 were targeted to identify novel drugs that could disrupt their interaction, thereby controlling the evasion of the human immune system mediated by ORF6. Furthermore, the molecular dynamics simulation technique was used to check the stability of drug-ORF6 complexes.

## Materials and methods

### Sequence retrieval and Mutation identification

The sequence of SARS-CoV-2 ORF6 protein (ID: P0DTC6) and the crystal structure of KPNA2 (PDB ID: 1EFX) protein were retrieved from UniProt online database (https://www.uniprot.org/) (19, 20). To detect single nucleotide substitutions in the ORF6 protein, we uploaded the sequence in FASTA format to the GISAID database (https://www.gisaid.org/). By comparing the submitted sequence with the reference sequence hCoV-19/Wuhan/WIV04/2019 (accession no MN996528.1), the server identified novel mutations and provided information about the positions of the substituted amino acid residues (21).

### 3D structure modeling and validation

The function of a protein is determined by its three-dimensional (3D) structure, which influences its interactions with other molecules in the body. In order to obtain the 3D structure of ORF6, the protein’s sequence was submitted to the Robetta server (https://robetta.bakerlab.org/) for structural modeling. The Robetta server utilizes Continuous Automated Model Evaluation (CAMEO) and has consistently demonstrated high precision and reliability since 2014 (22). To assess the quality of the modeled protein structure, it was subsequently subjected to validation tools, namely ProSa-Web (https://prosa.services.came.sbg.ac.at/prosa.php/) (23) and PDBsum (http://www.ebi.ac.uk/thornton-srv/databases/pdbsum/) (24). These online tools analyze the protein structure based on various quality scores.

### Structure and sequence-based protein stability analysis

To accurately predict the effects of mutants on protein stability, the mCSM server was employed, which utilizes a graph-based signature approach (http://biosig.unimelb.edu.au/mcsm/). For every single mutation, ΔΔG and RSA (relative solvent accessibility) values were calculated (25). For predicting the effect of alteration on dynamics and protein stability, through the NMA (Normal-Mode Analysis) approach, DynaMut2 (http://biosig.unimelb.edu.au/dynamut2) server was utilized. The aforementioned servers required a 3D structure of protein and mutations list for predicting mutational impact on protein structural stability. The ΔΔG (Gibbs free energy) value was estimated, the value less than zero (ΔΔG< 0.0 kcal/mol) shows destabilization however, the value greater than zero (ΔΔG > 0.0kcal/mol) shows stabilization (26). Furthermore, to find the effect of mutation on the structural stability based on protein sequence, we used the I-Mutant2.0 (http://folding.biofold.org/i-mutant/i-mutant2.0.html) server. The server needs a modified protein sequence and wild-type (WT) residue position to find the consequences of the exchange of amino acids on protein. Positive Gibbs free energy (ΔΔG) signifies high stability while negative ΔΔG signifies low stability (27).

### Variant modeling and superimposition

The wild-type structure of ORF6 underwent a minimization process, which aims to lower the energy of the protein structure. This procedure was performed using Chimera software, a molecular graphics and modeling program developed by the University of California, San Francisco (28). Additionally, the same software was utilized to model the highly deleterious and destabilizing mutations predicted in the wild-type ORF6 protein structure. Afterward, to check the structural variances between the WT and variants protein, PyMOL software was utilized to superimpose each mutant on the WT ORF6 structure and calculated the RMSD (root mean square deviation) value.

### Protein-protein docking and binding free energies calculation

To check the effect of mutations on the binding affinity of ORF6 with the KPNA2, we performed molecular docking by using the HDOCK server (29). This server uses the hybrid algorithm of template bases modeling and ab initio free docking and provides the top ten complex models with the highest scores. The scoring is based on an empirical potential made up of docking score and Ligand RMSD, with Vander Waals energy playing a minor role. For each interaction, the top-rank model was selected on the basis of a lower energy score (30). To visualize the results of interactions such as salt bridges, non-bonded contacts, and hydrogen bonds, the PDBsum online server was utilized. To determine the binding free energies of both wild-type and mutant ORF6 complexes, we employed the MM/GBSA approach. This approach is known for providing dependable estimates of binding free energies for a wide range of biological complexes (31). The calculation of binding free energies was carried out using the MMGBSA.py script, which considers contributions from electrostatic interactions, van der Waals forces, solvent-accessible surface area (SA), and generalized Born model (GB).

The following equation was used to calculate the binding free energies:


“ΔG(bind)=ΔG(complex)−[ΔG(receptor)+ΔG(ligand)]

To calculate each component of the total free energy individually, we employed the following equation:


“G=Gbond+Gele+GvdW+Gpol+Gnpol”

### Virtual drug screening against the binding interface of ORF6 with KPNA2

African natural product database was downloaded in 3D-SDF format (3D-structure data file), from the ANPDB website (African-natural product databases) (http://african-compounds.org/anpdb/) (31). ANPDB is an accumulation of medicinally important natural compounds. Before the screening of these databases, the FAF-Drugs 4 web server was used to get only drug-like non-toxic molecules that follow Lipinski’s rule of five (32). Subsequently, filtered databases were screened against the binding interfaces of the ORF6-KPNA2 complex. Before the screening, the drugs were changed to pdbqt format. AutoDock Vina was utilized for virtual drug screening, to screen the best drug-like molecules. Initially, 16 exhaustiveness was used for fast screening, after that 64 exhaustiveness was used for screening to reassess the best compounds and to eliminate false-positive results. For IFD (induced fit docking) the top 10% of drugs were selected from each database and screened by utilizing AutodockFR, which assists in covalent docking and facilitates receptor flexibility (33). Subsequently, the best final hits were processed for MD simulation analysis.

### Molecular dynamics simulation

The Amber20 package was used for molecular dynamics (MD) simulation to examine the top-hit drugs and ORF6 complexes’ stability (34) using the antechamber force field (35). TIP3P was utilized for the solvation of each system, and for system neutralization counter ions were added (36). MD simulation was carried out in several steps such as energy minimization, heating, equilibrium step, and production step. After neutralization, for bad clashes elimination the protocol of energy minimization was utilized that consists of 9000 cycles, first 6000 cycles use the steepest descent minimization (37) while the rest 3000 cycles use conjugate gradient minimization (38). Subsequently, the system was heated up to 300K and then equilibrated the system at constant pressure (1atm). Afterward, for 100 ns production step was run. Long-range electrostatics integrations were detected through the particle mesh Ewald method (34, 39). SHAKE algorithm was utilized to treat the Covalent bonds (40). Molecular dynamics simulation and trajectories were performed by PMEMD.CUDA and Amber20 CPPTRAJ packages, respectively. In the analysis of the complexes formed by the top hits and ORF6, the CPPTRAJ and PTRAJ packages were employed. These packages were utilized to examine the dynamic stability, compactness, and hydrogen bonding network of the complexes (41). To assess the structural dynamic stability, the Root Mean Square Deviation (RMSD) was computed. The RMSD value was determined by employing the mathematical formula below.


$$RMSD=\sqrt{\frac{\sum_{i=0}^{N}\left[m_{i}*{\left(X_{i}-Y_{i}\right)}^{2}\right]}{M}}$$

However, the Rg (radius of gyration) was employed to calculate the structural compactness.

## Results and discussion

**Figure 1:**
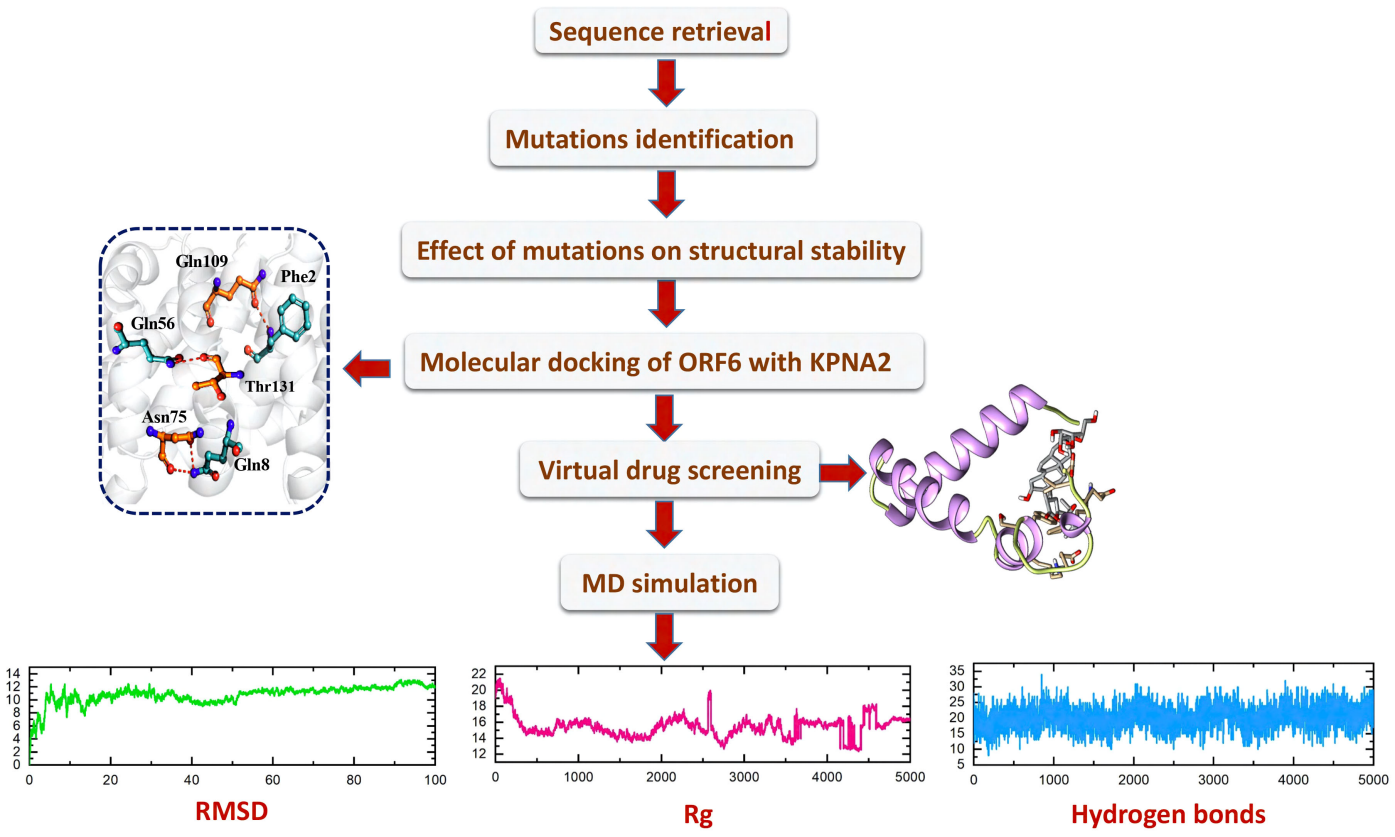
The overall workflow, including step-wise approaches used in this study.

With the passage of time, SARS-CoV-2 has undergone various mutations and become a more pathogenic and infectious strain until now (42). Mostly these mutations take place in the accessory, non-structural, and structural proteins, and have a direct impact on the infectivity, severity, and clinical outcomes of the virus (43). However, some proteins show resistance to mutation while others are prone to mutations (44). Among the accessory proteins of SARS-CoV-2, ORF6 was found to be the most pathogenic and mutational-prone protein. Multiple research studies have indicated that ORF6 plays a role in evading the human immune response by interacting with the KPNA2 complex, thereby antagonizing the interferons (IFNs) pathway (17, 18). As different mutants of ORF6 are continually emerging, it is crucial to investigate whether these mutants undermine IFN production and enhance the pathogenesis of viral infections by altering the stability of ORF6’s structure and its binding affinity with KPNA2. Consequently, this study was designed to examine the impact of recently identified mutations on the binding interaction between ORF6 and its target protein KPNA2 in the immune evasion pathway. Additionally, the study aimed to identify potential drugs that could target the binding interface of ORF6-KPNA2 and potentially mitigate the immune evasion properties of SARS-CoV-2. The overall workflow is shown in the **Figure 1**.

### Identification of newly emerged mutations in ORF6 protein

**Figure 2:**
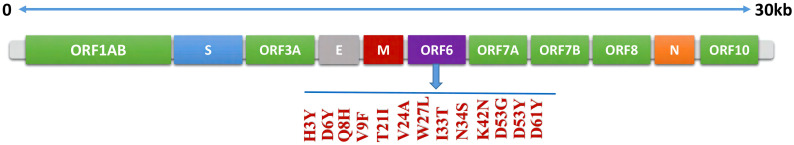
Schematic representation of mutations identified in ORF6 protein.

To identify the newly emerged mutations in the ORF6 protein, the sequence of ORF6 (ID: PODTC6) was retrieved from the UniProt database (19, 20) and submitted to the online database GSAID. The aforementioned database identified the newly emerged mutations in the ORF6 protein sequence by comparing it with the human coronavirus strain reported at Wuhan, China. The novel strain consists of 13 mutations (H3Y, D6Y, Q8H, V9F, T21I, V24A, W27L, I33T, N34S, K42N, D53G, D53Y, D61Y) existed on the ORF6 protein. the graphical representation of identified mutations is shown in **Figure 2**.

### Impact of mutation on the structural stability of ORF6 protein

The stability of a protein is the primary factor that affects the function, structure, and regulation of the protein (45). Mutation in the corresponding protein mainly affects their stability and can also cause protein malfunction. Mutation such as amino acid substitution promptly disrupts protein interaction with other bio-molecules, and can also affect protein fold, dynamics, function, and stability (46, 47). For understanding the role of mutation in causing disease the prediction of dynamics and stability of a protein is significant. Gibbs free energy (ΔΔG) stimulated by mutations was predicted, for the estimation of changes in the stability of protein upon mutation (26). Various computational approaches have been developed to estimate the mutational impact on protein stability by using protein structural or sequence information (48, 49). In the current study, various online servers such as DynaMut2, mCSM, and I-Mutant 2.0 were used for the prediction of protein functional and structural stability that alter upon mutation. Analysis of 13 variants through I-mutant 2.0 online server determined the ΔΔG value ranging from 1.73 kcal/mol to -2.36 kcal/mol, whereas six mutations (N34S, D53G, D53Y, D6Y, D61Y, H3Y) increase structural stability while seven mutations (K42N, V9F, Q8H, I33T, W27L, V24A, T21I) decrease structural stability (**Table 1**). These mutations were also analyzed by DynaMut2 server for further estimation of destabilizing variants. Analysis of 13 mutations through DynaMut2, it has been observed that ΔΔG value ranges from 1.49 kcal/mol to -1.39 kcal/mol. Out of thirteen variants, seven mutations (N34S, V9F, Q8H, I33T, W27L, V24A, T21I) were found to have a destabilizing effect on the structure of ORF6 protein while six mutations (K42N, D53G, D53Y, D6Y, D61Y, H3Y) have stabilizing effect, that enhance the stability of protein structure (**Table 2**).

**Table 1:** List of newly emerged mutations in ORF6, analyzed by DynaMut2 and I-Mutant 2.0.

Index	Variants	DynaMut2		I-Mutant 2.0	
		Predicted ΔΔG	Outcome	Predicted ΔΔG	Outcome
1	N34S	-0.48	Destabilizing	0.85	Stabilizing
2	K42N	1.14	Stabilizing	-1.29	Destabilizing
3	D53G	0.53	Stabilizing	0.38	Stabilizing
4	V9F	-0.75	Destabilizing	-2.36	Destabilizing
5	D53Y	0.15	Stabilizing	0.92	Stabilizing
6	D6Y	0.35	Stabilizing	0.3	Stabilizing
7	D61Y	0.12	Stabilizing	0.94	Stabilizing
8	H3Y	1.49	Stabilizing	1.73	Stabilizing
9	Q8H	-0.09	Destabilizing	-1.9	Destabilizing
10	I33T	-1.39	Destabilizing	-1.7	Destabilizing
11	W27L	-0.33	Destabilizing	-0.95	Destabilizing
12	V24A	-1.25	Destabilizing	-1.74	Destabilizing
13	T21I	-0.22	Destabilizing	-1.12	Destabilizing

**Table 2:** A list of mutations, analyzed by mCSM server to identify highly destabilizing mutations based on ΔΔG value.

Index	Variants	ΔΔG mCSM	Outcome
1	N34S	-0.871	Destabilizing
2	V9F	-1.143	Destabilizing
3	Q8H	-0.632	Destabilizing
4	I33T	-1.504	Destabilizing
5	W27L	-1.18	Destabilizing
6	V24A	-1.253	Destabilizing
7	T21I	-0.074	Destabilizing

Furthermore, to narrow down the list of highly destabilizing mutations identified by the DynaMut2 and I-Mutant 2.0 were further analyzed by mCSM. Among the destabilizing mutations, the variants such as V9F with ΔΔG value of -1.143 kcal/mol, I33T with ΔΔG value of -1.504 kcal/mol, W27L with ΔΔG value of -1.18 kcal/mol, and V24A with ΔΔG value of -1.253 kcal/mol, were reported as highly destabilizing variants that affect the structural stability of ORF6 protein. Although, mutations such as N34S, Q8H, and T21I with ΔΔG values of -0.871, -0.632, and -0.074 kcal/mol respectively, were reported as destabilizing variants that influenced minute changes in protein structure (**Table 2**). Similar approaches were used by several previous studies for the selection of highly destabilizing mutations (50, 51). To check the significance of these highly destabilizing mutations in human immune evasion, we further processed it to check its effect on the binding network of ORF6 and KPNA2.

### Variants Modeling of ORF6 protein and its superimposition on WT ORF6

**Figure 3:**
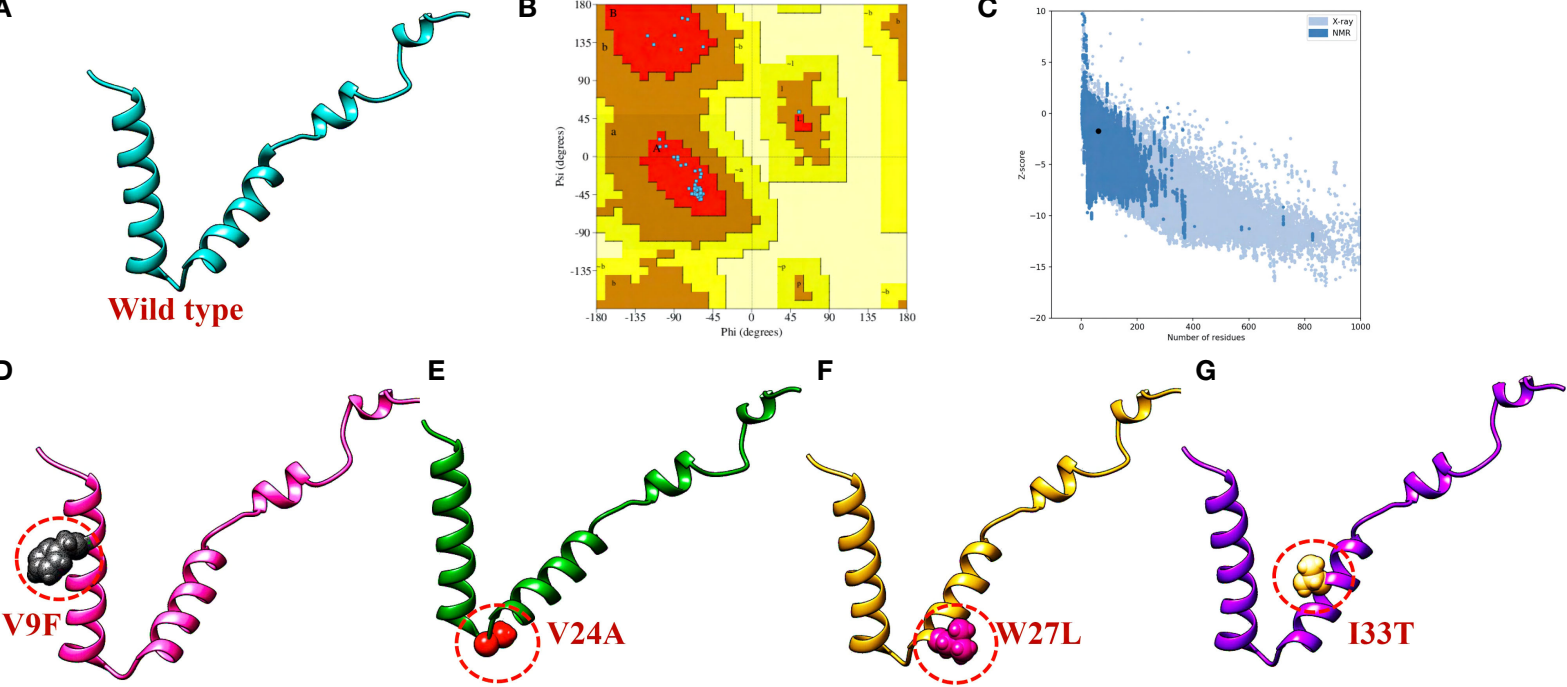
ORF6 3D structure validation and mutant modeling. **(A)** Showing wildtype ORF6, **(B)** validation by Ramachandran plot. **(C)** validation by ProSA-web **(D)** showing V9F mutant, **(E)** showing V24A mutant, **(F)** showing W27L mutant. **(G)** showing I33T mutant.

The three-dimensional (3D) structure of a protein plays a crucial role in determining its function and how it interacts with other molecules in the body. To determine the 3D structure of the ORF6 protein, its amino acid sequence was submitted to the Robetta server (https://robetta.bakerlab.org/) for structural modeling (**Figure 3A**). This server takes the protein sequence as input and generates five different models (22). To identify the best model among the generated structures, we utilized validation tools such as ProSa-Web (23) and PDBsum (24). First, the protein structures were subjected to Ramachandran analysis, and selected the model with the highest percentage of residues in the favorable region and the fewest outliers (**Figure 3B**). This selection process ensured that the chosen model exhibited a conformation that was most likely to be biologically relevant and structurally sound. Moreover, we employed the ProSA-web tool to assess the quality of the best models and identify any potential errors. The resulting Z score from ProSA-web analysis was -1.73, which falls within the range expected for normal protein structures of similar size (52) (**Figure 3C**). Subsequently, to assess how selected destabilizing mutations (V9F, V24A, W27L, I33T) influenced the binding affinity between ORF6 and KPNA2, we incorporated these mutations into the wild-type ORF6 protein using Chimera software for modeling (**Figures 3D–G**).

**Figure 4:**
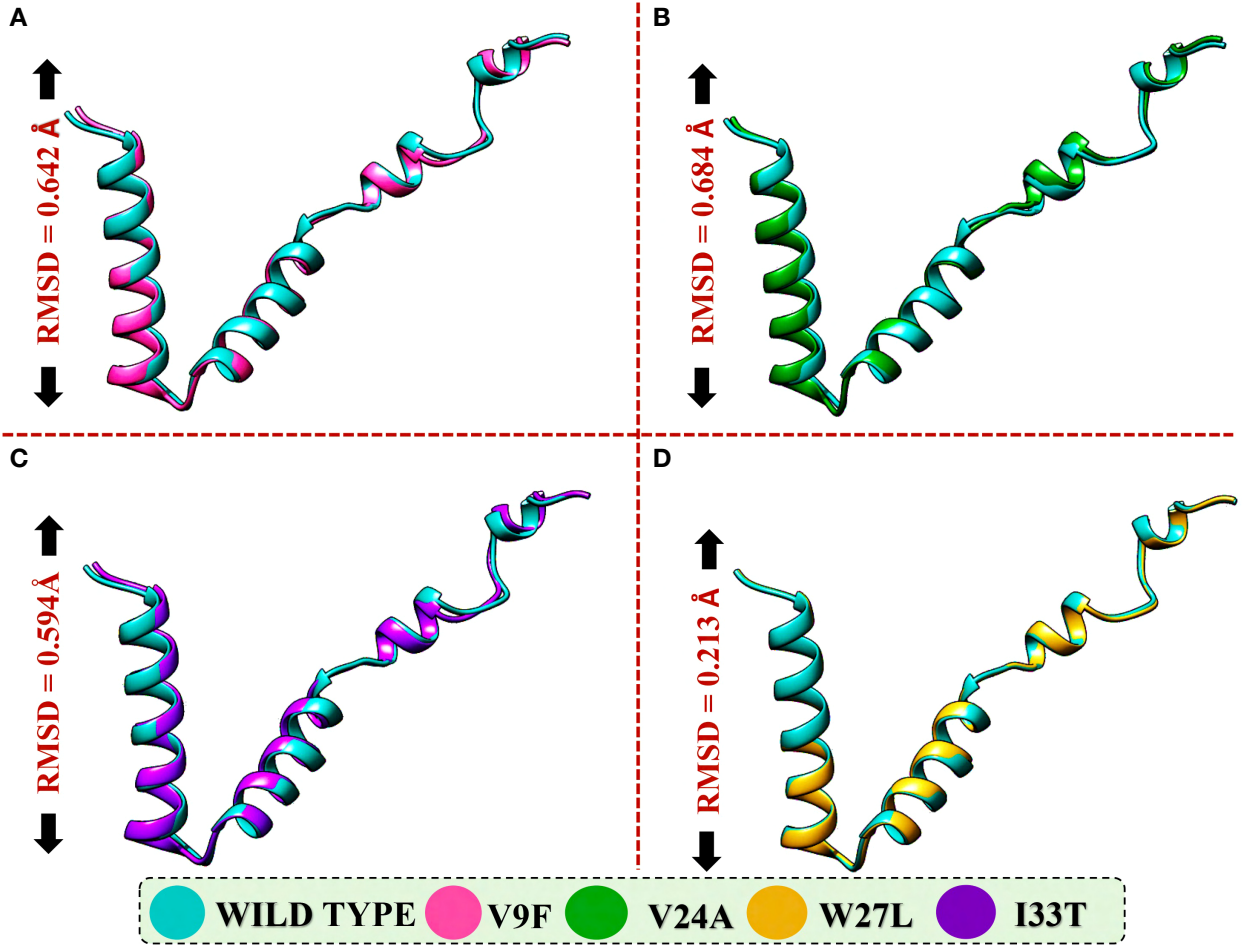
Superimposition of WT ORF6 with ORF6 mutants. **(A)** Showing RMSD value of V9F, **(B)** showing RMSD value of V24A, **(C)** showing RMSD value of W27L, **(D)** showing RMSD value of I33T.

To evaluate the structural differences between the generated mutants and the wild-type ORF6 protein, their respective structures were superimposed, and the root-mean-square deviation (RMSD) values were calculated (**Figure 4**). The RMSD values indicated significant differences between the mutants and the wild-type protein, with values of 0.64 Å, 0.68 Å, 0.59 Å, and 0.21 Å for the V9F, V24A, W27L, and I33T mutants, respectively. The identified mutations led to changes in the protein’s secondary structure and conformation, highlighting the significance of examining how they might affect the binding affinity between ORF6 and KPNA2. Subsequently, we utilized molecular docking, a structural methodology to investigate the influence of these mutants on the binding affinity of ORF6 with KPNA2.

### Bonding network analysis of wildtype and mutant ORF6 and KPNA2

**Figure 5:**
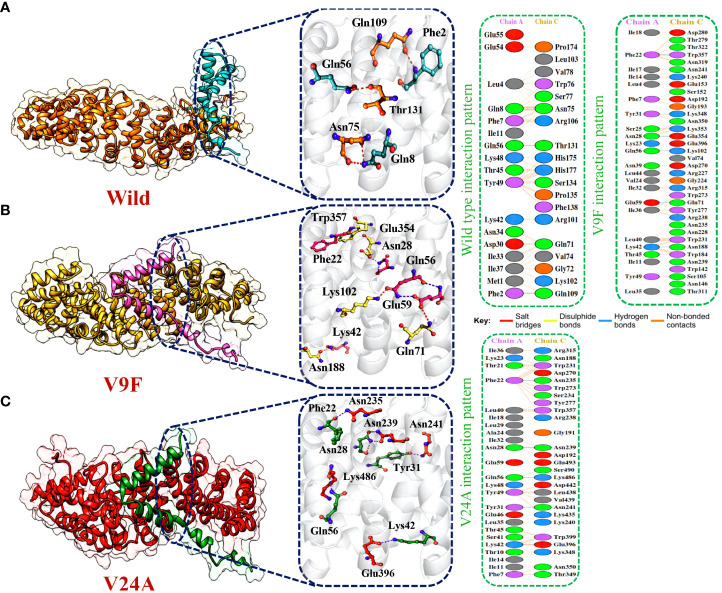
Bonding network analysis of wildtype and mutant ORF6-KPNA2 complexes. **(A)** Represents the wildtype-KPNA2 bonding network. **(B)** Represents the V9F-KPNA2 bonding network. **(C)** Represents the V24A-KPNA2 bonding network.

The application of molecular docking in studying protein-protein interactions (PPIs) has proven valuable in understanding the structure and function of PPIs in disease progression. By utilizing molecular docking techniques to predict the binding modes and conformations of proteins involved in PPIs, researchers can gain valuable insights into the underlying mechanisms of disease progression (53). ORF6 protein has a key role in the evasion of the human immune system. ORF6 protein physically binds with KPNA2 to inhibit IRF3 and STAT1 nuclear translocation and hence antagonize IFN production. Due to the importance of ORF6 and KPNA2 in immune evasion and regulating IFN signaling and production pathways, binding analysis for ORF6 WT and its various mutants with KPNA2 was performed (54). For regulation and understanding of these biological processes, the crucial steps are binding efficiencies and structural determination of the particular interactions. Significantly, binding affinity, which regulates molecular interactions, discovers whether the complex formation takes place under certain circumstances (55). To determine the structural mechanisms of higher pathogenicity of various mutants of SARS-CoV-2, molecular docking of KPNA2 with WT ORF6 and its various mutants including V9F, V24A, W27L, and I33T was performed by using the HDOCK server. For the wild type ORF6-KPNA2 complex, the HDOCK predicted docking score was recorded to be -253.72 kcal/mol. Interaction interface analysis by PDBsum showed that the complex formed 153 non-bonded contacts and 4 hydrogen bonds. The residues that formed hydrogen bonds between the WT ORF6-KPNA2 complex were Gln56-Thr131, Phe2-Gln109, and Gln8-Asn75 (**Figure 5A**). However, the predicted docking score for the V9F-KPNA2 complex was -267.90 kcal/mol. The PDBsum analysis showed the formation of 224 non-bonded contacts and 5 hydrogen bonds between the binding interface of ORF6 and KPNA2. The key residues Phe22-Trp357, Asn28-Glu354, Gln56-Lys102, Glu59-Gln71, Lys42-Asn188 formed the hydrogen bonds between KPNA2 and V9F variant (**Figure 5B**). Furthermore, the docking score of -258.41kcal/mol was predicted for V24A-KPNA2 complex, while the analysis of the binding interface by PDBsum revealed the presence of 5 hydrogen bonds, 1 salt bridge, and, 176 non-bonded contacts. The residues Phe22-Asn235, Asn28-Asn239, Gln56-Lys486, Tyr31-Asn241, and Lys42-Glu396 formed the hydrogen bonds while the residues Lys42-Glu396 formed a salt bridge between KPNA2 and V24A mutant (**Figure 5C**). The results demonstrated that mutants increased the binding affinity of ORF6 with KPNA2 as compared to the wild type, hence may further accelerate ORF6 protein function to evade host immune response.

**Figure 6:**
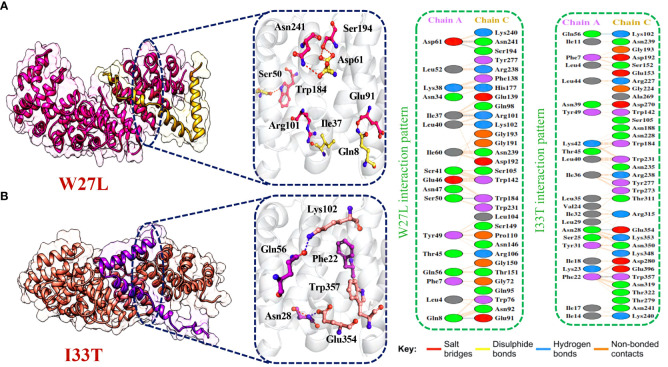
Bonding network analysis of W27L and I33T ORF6-KPNA2 complexes. **(A)** Represents the W27L-KPNA2 bonding network. **(B)** Represents the I33T-KPNA2 bonding network.

Afterward, HDOCK predicted a docking score of -254.51 kcal/mol for the W27L-KPNA2 complex. PDBsum analysis showed 156 non-bonded contacts and 5 hydrogen bonds. The key residues Asp61-Asn241, Asp61-Ser194, Ile37-Arg101, Ser50-Trp184, Gln8-Glu91 formed the hydrogen bonds between KPNA2 and W27L variant (**Figure 6A**). Finally, for the I33T-KPNA2 complex, HDOCK predicted a docking score of -268.79 kcal/mol, and PDBsum analysis revealed the existence of 3 hydrogen bonds and 239 non-bonded contacts. Between KPNA2 and I33T variant, the residues Gln56-Lys102, Asn28-Glu354, and Phe22-Trp357 formed hydrogen bonds (**Figure 6B**). The docking results indicated that these selected highly destabilizing mutants significantly increased the binding affinity of ORF6 and KPNA2 as compared to the wildtype complex, which may enhance the ability of SARS-CoV-2 to evade the human immune system. Utilizing the docking score and analysis of hydrogen bonding networks, it was established that among the examined mutants, the V9F variant displayed the most substantial binding affinity with KPNA2. As a result, we opted to concentrate our subsequent analysis on this specific mutant for the purpose of screening potential drugs. Our focus on the V9F mutant aimed to explore its viability as a target for drug discovery and development concerning ORF6-KPNA2 interactions.

### Binding free energies calculation by MM/GBSA approach

Binding free energy calculations are commonly employed to accurately assess the binding strength and structure of small molecules. This calculation plays a vital role in enhancing the precision and dependability of docking predictions, surpassing conventional docking and alchemical methods (56). The approach is widely used to investigate the interaction potency and uncover key binding properties that govern the overall binding mechanism (57). Consequently, we employed the MM/GBSA approach to evaluate the overall binding energy of complexes formed by the wild type and mutant ORF6-KPNA2. As shown in **Table 3** the recorded Van der Waals energies: -96.39 kcal/mol for the wild-type complex, -153.43 kcal/mol, -132.63 kcal/mol, -121.63 kcal/mol, and -156.96 kcal/mol for the V9F, V24A, W27L, and I33T mutants, respectively. In terms of electrostatic energies, the estimates were 282.69 kcal/mol for the wild-type complex, -68.44 kcal/mol, 463.94 kcal/mol, 416.92 kcal/mol, and -54.34 kcal/mol for the V9F, V24A, W27L, and I33T mutants, respectively. The results for total binding free energies indicated -29.92 kcal/mol for the wild type, and -42.28 kcal/mol, -10.06 kcal/mol, -21.12 kcal/mol, and -39.04 kcal/mol for the V9F, V24A, W27L, and I33T mutants, respectively. These findings demonstrate that the mutant V9F exhibits the highest binding free energy, thereby confirming the results obtained from molecular docking.

**Table 3:** Binding free energies analysis of wildtype and mutant ORF6-KPNA2 complexes.

Complexes	vdW	Electrostatic	GB	SA	Total Binding Energy
Wild Type	-96.39	282.69	-203.97	-12.25	-29.92
V9F	-153.43	-68.44	199.78	-20.19	-42.28
V24A	-132.63	463.94	-323.41	-17.97	-10.06
W27L	-121.63	416.92	-300.09	-16.32	-21.12
I33T	-156.96	-54.34	192.85	-20.58	-39.04

### Drug screening analysis of V9F mutant of ORF6

Virtual drug screening is a valuable technique in the field of drug design as it enables researchers to identify and assess potential drug candidates before embarking on expensive and time-consuming laboratory experiments. This approach plays a crucial role in the drug design process by offering a faster and more efficient means of identifying potential drug candidates and optimizing their chemical and biological properties (58, 59). Prior to performing a database screening, the molecules underwent filtration using Lipinski’s rule of five to identify molecules with drug-like characteristics. AutoDock Vina was utilized for computational drug screening, against the binding interface of ORF6 and KPNA2. Among the 954 molecules, only 745 compounds passed the ADMET criteria. The first step of virtual screening reported that the docking score of the 745 compounds ranges from -6.2 to -1.0 kcal/mol. To conduct further analysis, compounds with a score below -5.0 kcal/mol were chosen. Based on this criterion, a total of 130 compounds were selected and subjected to induced fit docking, resulting in docking scores ranging from -6.4 to -2.9 kcal/mol. Among these compounds, the top 5 hits were selected based on their favorable interaction profiles and high docking scores. The docking scores of top 5 hits, namely 1,3,5-trihydroxy-6,7-dimethoxy -2,4-bis(4-methylpent-3-enyl) xanthen-9- one, (3R,5S)-3-[(3S,5S,9R,10S,13S,14R,16S,17R)-16-hydroxy-10,13-dimethyl-3-[(2R,3S,4S,5R,6S)-3,4,5-trihyd, [(2S,3R,4R,5S,6S)-3,4,5 -trihydroxy-6-[2- (4-hydroxyphenyl) ethoxy]tetrahydropyran-2-yl]methyl, 3-(3,4- dihydroxyphenyl)-6,8-dihydroxy-2-[(2S,3R,4S,5R)-2,3,4-trihydroxy-5-methyl-tetrahydropyran-2-y, and 8-oxo-16-[(2R,3S,4S,5S,6R) -3,4,5- trihydroxy-6-(hydroxymethyl) tetrahydropyran-2-yl] oxy-hexadecanoic were -6.40 kcal/mol, -6.10 kcal/mol, -6.09 kcal/mol, -6.06 kcal/mol and -6.03 kcal/mol respectively. The top 5 compounds along with their drug names, 2D structures, and docking score are given in **Table 4**.

**Table 4:** List of top 5 hits along with their 2D structures, names and docking scores.

Top hit#	Drug Name	2D structure	Docking score
1	1,3,5-trihydroxy-6,7-dimethoxy -2,4-bis(4-methylpent-3-enyl) xanthen-9- one	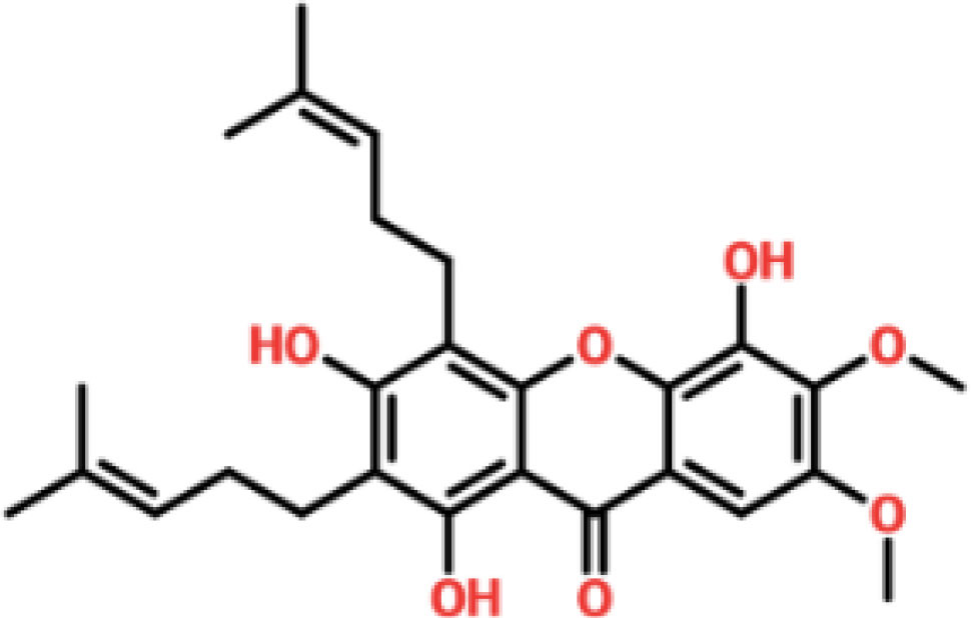	-6.40 kcal/mol
2	(3R,5S)-3-[(3S,5S,9R,10S,13S,14R,16S,17R)-16-hydroxy -10,13-dimethyl -3- [(2R,3S,4S,5R,6S)-3, 4,5-trihyd	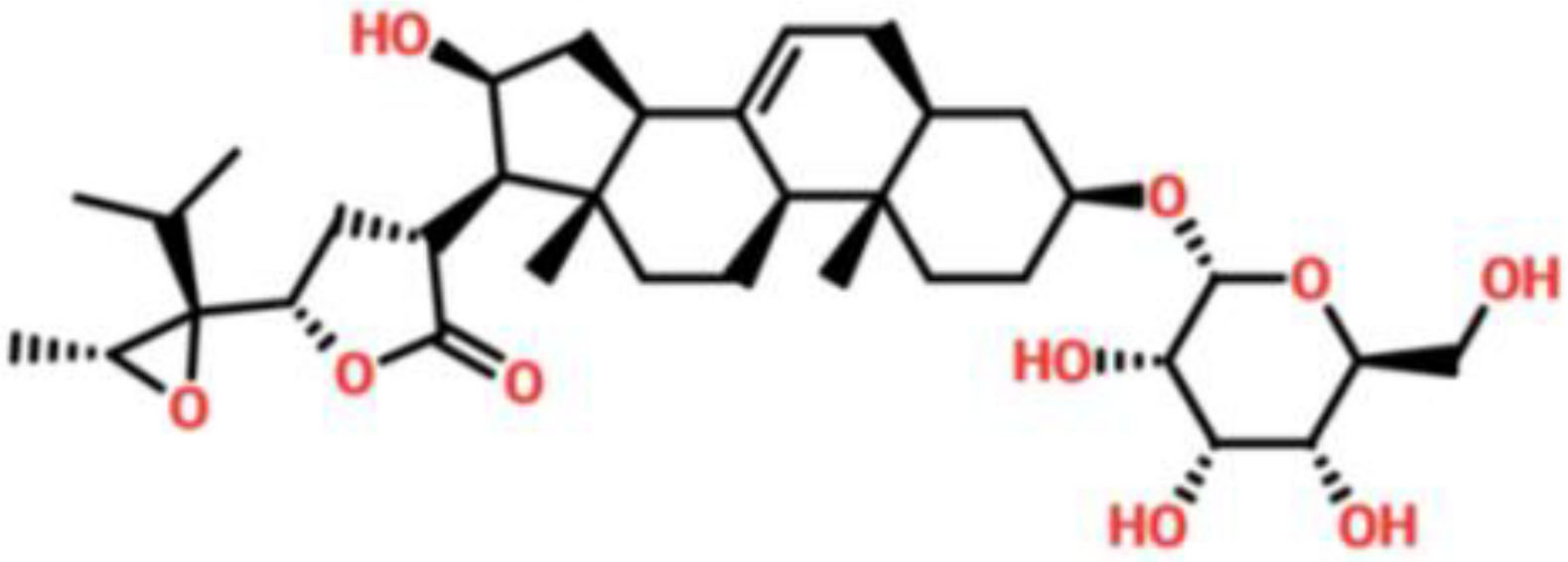	-6.10 kcal/mol
3	[(2S,3R,4R,5S,6S)-3,4,5 -trihydroxy -6-[2-(4-hydroxyphenyl)ethoxy]tetrahydropyran-2-yl]methyl	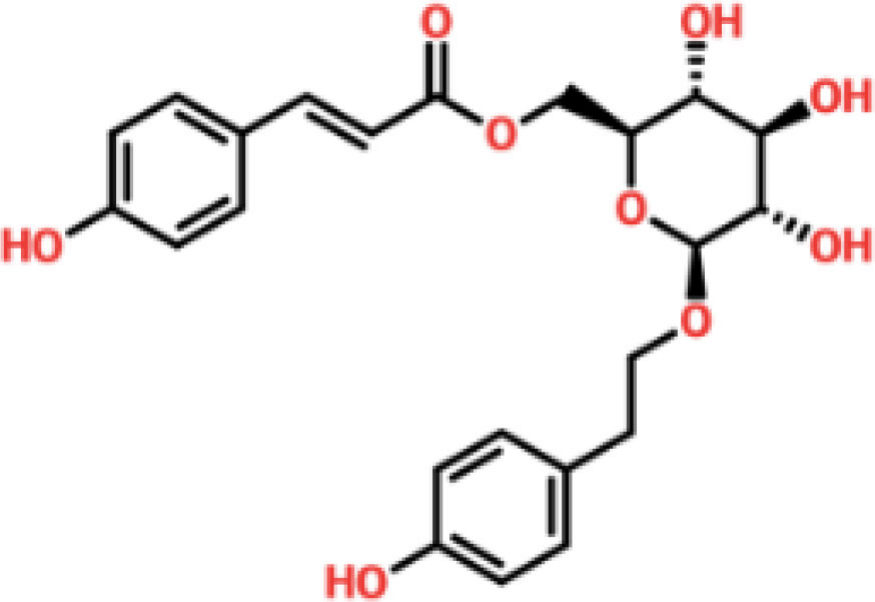	-6.09 kcal/mol
4	3-(3,4-dihydroxyphenyl)-6,8-dihydroxy-2-[(2S,3R,4S,5R) -2,3,4-trihydroxy -5-methyl- tetrahydropyran-2-y	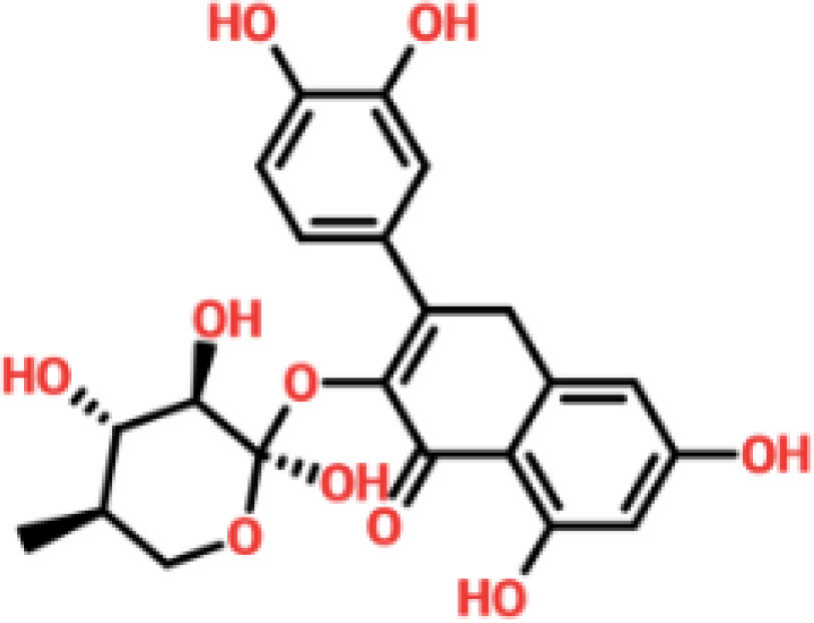	-6.06 kcal/mol
5	8-oxo-16-[(2R,3S,4S,5S,6R) -3,4,5 -trihydroxy -6-(hydroxymethyl) tetrahydropyran-2-yl] oxy-hexadecanoic	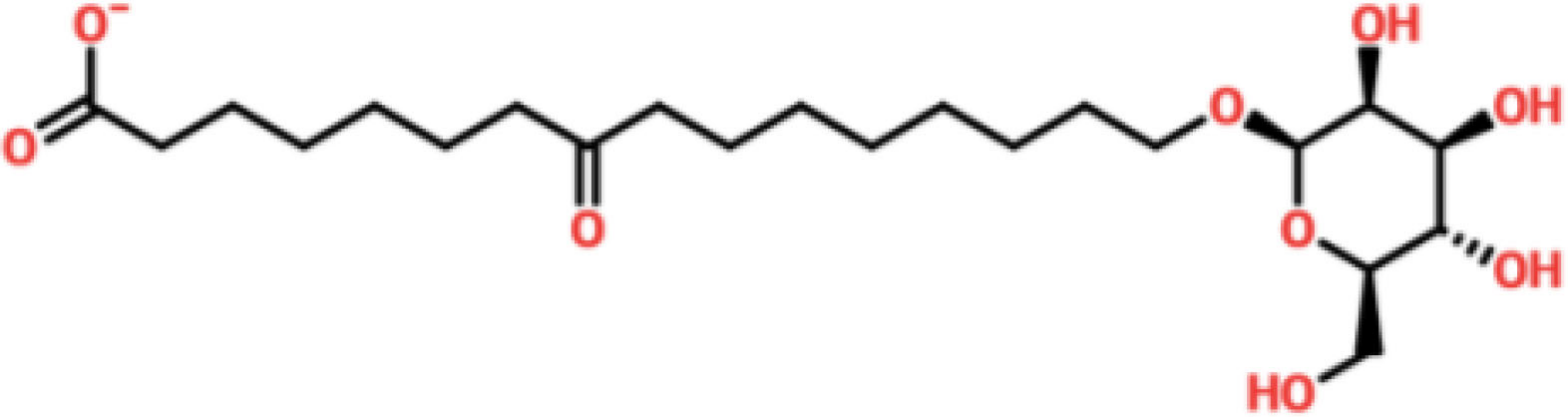	-6.03 kcal/mol

### Interaction analysis of top hits compounds

**Figure 7:**
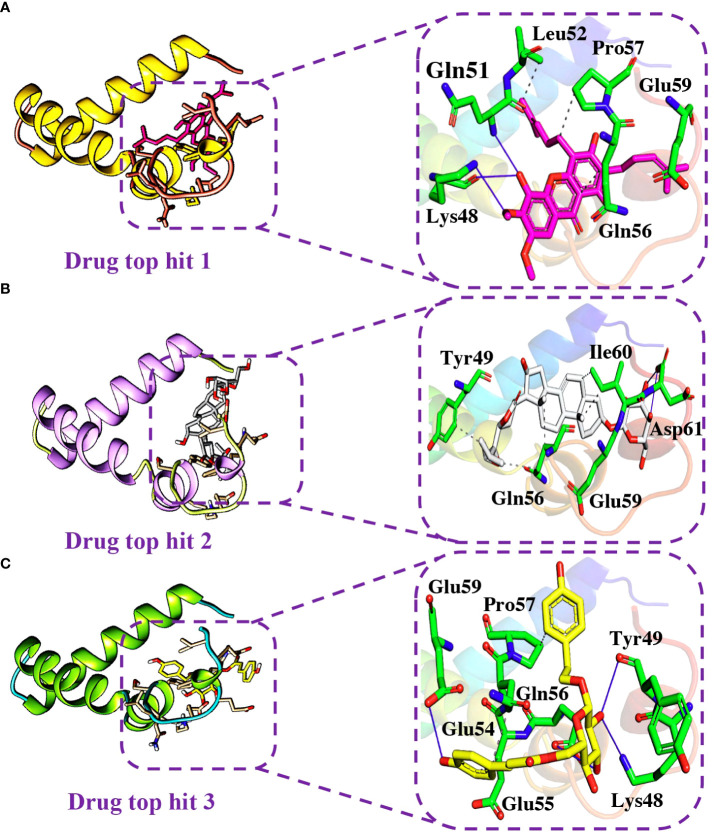
Binding modes of top hit 1, top hit 2, and top hit 3. **(A)** Showing hydrogen bonding network of top hit 1. **(B)** Showing hydrogen bonding network of top hit 2. **(C)** Showing hydrogen bonding network of top hit 3.

Detailed analysis of the top 5 hits gives information about hydrophobic interaction, hydrogen bonds, and salt bridges. In the case of tophit1-ORF6 complex, the bonding network analysis revealed the docking score of -6.40 kcal/mol with the formation of 4 hydrogen and 4 hydrophobic bonds with the specific residues in the target protein. The key residues Lys48, Gln51, Leu52, Gln56, Pro57, and Glu59 were involved in the bonding network formation (**Figure 7A**). Next, Interaction analysis of tophit2-ORF6 complex reported the formation of 4 hydrogen bonds with Tyr49, Gln56, Glu59, Ile60, and Asp61 residues. Additionally, the compound also showed the existence of 5 hydrophobic bonds (**Figure 7B**). The recorded docking score for tophit2-ORF6 complex was -6.10 kcal/mol. Furthermore, our bonding network analysis revealed that the tophit3-ORF6 complex exhibited favorable interactions with the target protein with 3 hydrophobic bonds and 4 hydrogen bonds with amino acids Lys48, Tyr49, Glu54, Glu55, Gln56, Pro57, and Glu59 however, the docking score for the aforementioned complex was recorded to be -6.09 kcal/mol (**Figure 7C**). In conclusion, the above three top hits targeted the important amino acid residues (Gln56, Glu59) which were involved in the interaction between ORF6 and KPNA2. Hence, these hits reported better pharmacological potential for exhibiting higher docking scores and better interaction paradigms.

**Figure 8:**
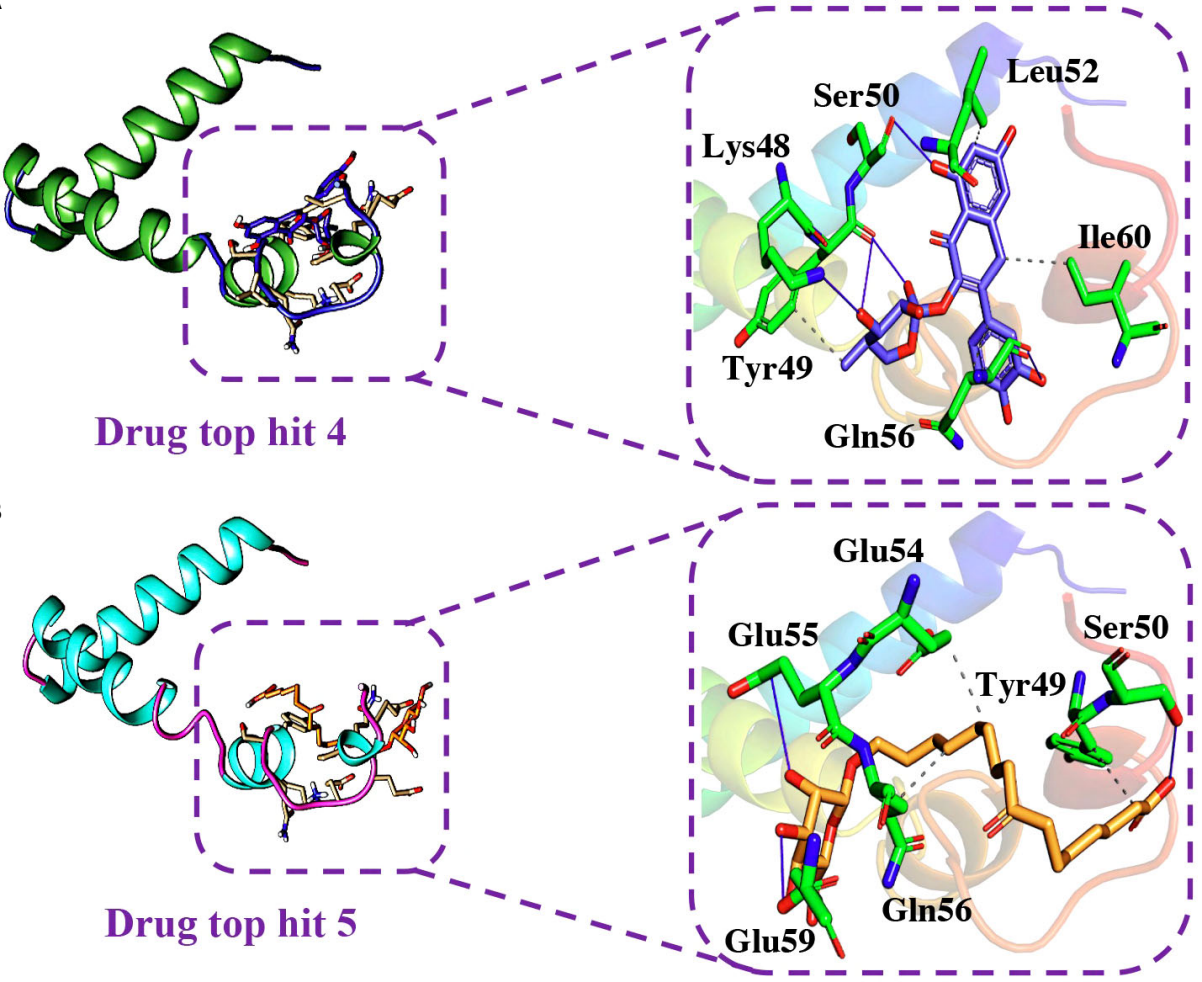
Binding modes of top hit 4 and top hit 5. **(A)** Showing hydrogen bonding network of top hit 4. **(B)** Showing hydrogen bonding network of top hit 5.

Similarly, the analysis of the tophit4-ORF6 complex showed the formation of 5 hydrogen bonds and 4 hydrophobic interactions with a docking score of -6.06 kcal/mol. The amino acid residues involved in the bonding network formation were Lys48, Tyr49, Ser50, Leu52, Gln56, and Ile60 (**Figure 8A**). Finally, the analysis of the tophit5-ORF6 complex revealed the formation of 3 hydrophobic bonds and 4 hydrogen bonds with a docking score of -6.03 kcal/mol. The key residues Tyr49, Ser50, Glu54, Glu55, Gln56, and Glu59 were involved in the bonding network formation in the target protein (**Figure 8B**). Our findings indicate that these compounds hold considerable promise as drug candidates due to their favorable interactions with specific amino acid residues (Ser50, Gln56, and Glu59) crucial in the interaction between ORF6 and KPNA2, potentially enhancing their therapeutic effectiveness. To assess the dynamic stability of the top hits-ORF6 complexes, we chose the top three drugs for molecular dynamic simulation analysis.

### Molecular dynamics simulations analysis of top hits

**Figure 9:**
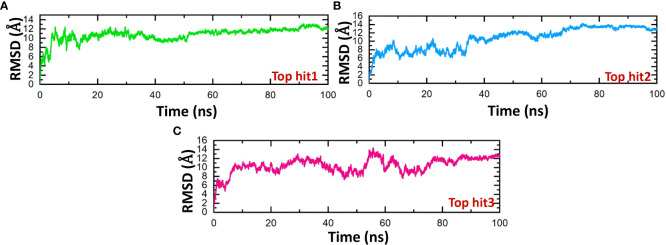
Dynamics stability analysis of drug-ORF6 complex. **(A)** Showing the RMSD value of hop hit 1. **(B)** Showing the RMSD value of hop hit 2. **(C)** Showing the RMSD value of hop hit 3.

The stability of molecular interactions within a binding cavity is a critical factor in finding the binding efficiency of small ligand molecules. To analyze this stability, simulation trajectories can be employed, and one metric that can be calculated is RMSD (root mean square deviation). This metric provides details on the dynamic stability of interacting molecules, which can shed light on the binding strength. Understanding a protein’s dynamic stability is crucial in estimating the stability of biological complexes in a dynamic environment (60). Therefore, we calculated the RMSD over the 100ns simulation to analyze the binding stability of drug-protein complexes. According to the RMSD values in **Figure 9**, the top hits 1-3 exhibited stable behavior during the 100ns simulations. The top hit 1 system equilibrated at 20ns and remained stable until the end of the simulation. The top hit 1 declared the most stable complex in terms of RMSD with no major convergence was observed (**Figure 9A**). In the case of a top hit 2, the system equilibrated at 5ns however the values of RMSD raised gradually until 60ns. In the top hit 2 system a little convergence was observed during 10ns and 40ns then the system remained stable until the end of the simulation (**Figure 9B**). In the case of top hit 3, the system gained stability at 10ns and remained stable until the end of the simulation, however a significant convergence was noted from 50ns to 70ns (**Figure 9C**). In summary, top hits 1-3 have stable dynamics and could bind strongly with interface residues to reduce the binding of ORF6 and KPNA2.

**Figure 10:**
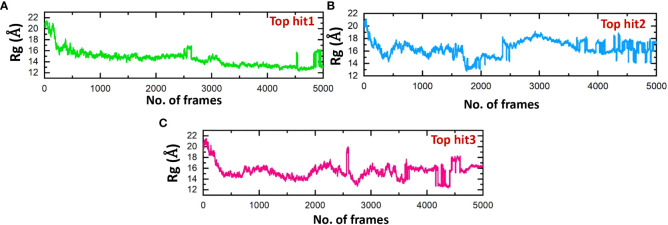
Compactness analysis of drug-ORF6 complex. **(A)** Showing the Rg value of hop hit 1. **(B)** Showing the Rg value of hop hit 2. **(C)** Showing the Rg value of hop hit 3.

The structure stability of every complex was examined in a dynamics setting to look into the occurrence of unbinding and binding events. This was accomplished by calculating Rg (radius of gyration), structural compactness measurement, over the time of 100ns. The previous studies showed that the protein complexes’ compactness was crucial to their stability (61). Comparing the results shown in **Figure 10** to the Root Mean Square Deviation reveals a similar pattern in terms of compactness. In the case of top hit 1, the Rg value showed stable behavior throughout the simulation time frame with no significant convergence. The average Rg value of 14 Å was recorded (**Figure 10A**). Likewise, the average Rg value of 16 Å was recorded for the top hit 2 system. In the case of top hit 2, a significant convergence was observed at various points during the simulation (**Figure 10B**). Finally, for top hit 3 the average Rg value was recorded to be around 15 Å however a little convergence was observed at the later stages of the simulation (**Figure 10C**). Changes in the Rg are indicative of unbinding and binding events between the receptor and ligands. These results strongly exhibit that top hits 1-3 have substantial pharmacological activity against ORF6.

**Figure 11:**
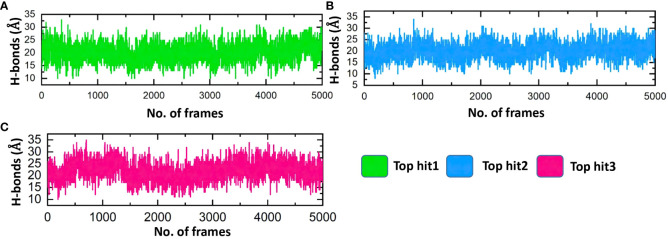
Bonding network analysis between top hit drugs and ORF6. **(A)** Showing hydrogen bonds numbers in top hit 1. **(B)** Showing hydrogen bonds numbers in top hit 2. **(C)** Showing hydrogen bonds numbers in top hit 3.

Assessing the hydrogen bonds formed during molecular interactions is a useful approach to evaluating binding affinity (62). It is essential to comprehend the bonding patterns of hydrogen involved in drug-protein interactions to predict the strength of these interactions accurately (63, 64). Throughout the simulation, the hydrogen bond numbers were determined for each trajectory to examine the evolution of the hydrogen bonding pattern. Each complex’s hydrogen bonding network was examined over time, and the outcomes are exhibited in **Figure 11**. **Figure 11** illustrates that the ligand-protein complexes formed a strong network of hydrogen bonds, indicating stable interactions between the top-hit drugs and ORF6. The average hydrogen bond numbers observed in the top three drug-ORF6 complexes were 23, 20, and 25, respectively (**Figures 11A–C**). These results support the findings from the molecular docking and RMSD analyses, providing further evidence of the stability of the complexes.

## Conclusion

In this study, we identified that the mutations V9F, V24A, W27L, and I33T have a substantial destabilizing effect on the structure of the ORF6 protein. Moreover, by conducting molecular docking analysis between the wildtype and mutant ORF6 and KPNA2, we observed that the V9F, V24A, W27L, and I33T mutations exhibited a stronger binding affinity with KPNA2 compared to the wildtype ORF6, Notably, the V9F mutation demonstrated the highest binding affinity, as supported by the calculated binding free energy of -42.26 kcal/mol. Consequently, these mutations could enhance the functionality of the ORF6 protein in evading the host immune response. To counteract this interaction, we employed molecular screening and simulation techniques to design novel inhibitors derived from natural products. Our findings identified five compounds as the most promising candidates based on favorable docking scores and binding stability. However, experimental validation is required to confirm their efficacy. Overall, this study represents the first step toward understanding the heightened infectivity of new SARS-CoV-2 variants and provides a strong rationale for the development of novel drugs targeting these variants.

## Data availability statement

The original contributions presented in the study are included in the article/supplementary material. Further inquiries can be directed to the corresponding author.

## Author contributions

AS: Data curation, Formal Analysis, Investigation, Methodology, Visualization, Writing – original draft. MS: Conceptualization, Methodology, Project administration, Supervision, Writing – original draft. HK: Investigation, Methodology, Validation, Writing – review & editing. SR: Data curation, Investigation, Methodology, Writing – review & editing. SC: Methodology, Project administration, Supervision, Validation, Writing – review & editing. HY: Conceptualization, Funding acquisition, Investigation, Project administration, Supervision, Writing – review & editing. AA: Writing – review & editing, methodology, validation, resources..
